# Nonsteroidal Anti-Inflammatory Drugs and the Kidney

**DOI:** 10.3390/ph3072291

**Published:** 2010-07-21

**Authors:** Walter H. Hörl

**Affiliations:** Division of Nephrology and Dialysis, Department of Medicine III, Medical University of Vienna, Währinger Gürtel 18-20, A-1090 Vienna, Austria; E-Mail: walter.hoerl@meduniwien.ac.at; Tel.: +43-1-40400 4390; Fax: +43-1-40400-4392

**Keywords:** non-steroidal anti-inflammatory drugs, cyclooxygenase, celecoxib, acute renal failure, chronic kidney disease

## Abstract

Non-steroidal anti-inflammatory drugs (NSAIDs) inhibit the isoenzymes COX-1 and COX-2 of cyclooxygenase (COX). Renal side effects (e.g., kidney function, fluid and urinary electrolyte excretion) vary with the extent of COX-2-COX-1 selectivity and the administered dose of these compounds. While young healthy subjects will rarely experience adverse renal effects with the use of NSAIDs, elderly patients and those with co-morbibity (e.g., congestive heart failure, liver cirrhosis or chronic kidney disease) and drug combinations (e.g., renin-angiotensin blockers, diuretics plus NSAIDs) may develop acute renal failure. This review summarizes our present knowledge how traditional NSAIDs and selective COX-2 inhibitors may affect the kidney under various experimental and clinical conditions, and how these drugs may influence renal inflammation, water transport, sodium and potassium balance and how renal dysfunction or hypertension may result.

## 1. Introduction

Nonsteroidal anti-inflammatory drugs (NSAIDs) represent one of the most common classes of medications used world-wide, with an estimated usage of >30 million per day [1]. NSAIDs exert anti-inflammatory, analgesic and anti-pyretic effects through the suppression of prostaglandin (PG) synthesis, by inhibiting the enzyme cyclooxygenase (COX). Two isoforms of this enzyme COX-1 and COX-2 are known to exist. The gastrointestinal tract and the kidneys are important targets for untoward clinical events associated with the use of NSAIDs [2]. Approximately 2.5 million Americans experience NSAID-mediated renal effects yearly [3].

Nonselective NSAIDs inhibit both COX-1 (expressed constitutively in the kidney) and COX-2 (inducible in most tissues in response to injury or inflammation, but also present at detectable levels in normal adult mammalian kidneys), the rate limiting enzymes for the production of PGs and thromboxane (TX) [4]. COX-2 is regulated in response to intravascular volume [5]. COX-1 functions mainly in the control of renal hemodynamics and glomerular filtration rate (GFR), while COX-2 functions primarily affect salt and water excretion [6]. Blockade of either or both of these enzymes can have, therefore, different effects on renal function [7,8].

## 2. Physiology and Pathophysiology of COX Inhibition

PGs regulate a wide variety of renal functions [9]. PGE_2_ is considered to be mainly a tubular PG and PGI_2_ a vascular PG. However, renal arterioles, tubules, medullary interstitial cells, and mesangial cells are able to produce both PGE_2_ and PGI_2_. PGE_2_ regulates sodium and chloride transport in the loop of Henle and modulates water transport and renal medullary blood flow. The physiological effects of PG_2_ are mediated through the four G-protein-coupled transmembrane prostaglandin receptors EP1, EP2, EP3 and EP4. PGI_2_ regulates renal vascular tone, GFR and renin release [10]. In a person with normal renal hemodynamic parameters, PGs do not play a dominant physiologic role in maintaining renal blood flow and GFR [11]. Selective COX-2 inhibitors were developed to produce the beneficial effects of NSAIDs, but spare the COX-1-mediated adverse events [12]. However, COX-2 appears to be associated with renal vascular tissues and podocytes and has been implicated as the dominant COX at the macula densa and in the medullary interstitium. The identification of constitutive COX-2 in the human kidney [13], and the recognition of the profound effects of PGs on renal homeostasis [14] may indicate that COX-2 inhibitors have the same potential for adverse renal effects as traditional NSAIDs [12]. Therefore, the same precautions in patients at risk for adverse renal effects probably apply to both the nonselective NSAIDs and COX-2 selective inhibitors [4].

In normotensive subjects neither blood pressure nor renal function is significant affected by selective COX-2 inhibitors or nonselective NSAIDs [15,16]. In contrast, inhibition of PG synthesis leads to renal decompensation in situations where renal and systemic hemodynamics are dependent on the availability of PGs [10]. In salt-depleted healthy subjects, selective inhibition of COX-2 causes sodium and potassium retention [17,18,19]. In elderly patients with compromised renal function, selective COX-2 inhibitors and nonselective NSAIDs may cause reductions in GFR and a reduction in urinary sodium excretion, urinary PGE_2_, and 6-keto-PGF_1α_ excretion [20,21]. In elderly subjects with hypertension, treatment with COX-2 selective inhibitors may promote edema formation and elevations in blood pressure [22,23]. Patient groups who are at risk for renal adverse effects from NSAIDs include those with extreme liver dysfunction, or those with nephrotic syndrome and high-level proteinuria, or those with very low renal function [24]. 

In volume-depleted rats obtained by oral furosemide, treatment with the anti-inflammatory selective COX-2 inhibitor flosulide or indomethacin caused a decrease in renal plasma flow and GFR, and also a fall in urinary 6-keto-PGF_1α_ and TXB_2_ excretion, indicating that not only traditional NSAIDs but also selective cyclooxygenase inhibitors (coxibs) alter renal function in hypovolemic animals [25]. In humans, acute renal failure (ARF) in the setting of NSAID administration may occur in situations where the kidney is more dependent on PGs in the maintenance of renal blood flow and GFR, such as congestive heart failure, nephrotic syndrome, liver cirrhosis and salt depletion [10].

Liver cirrhosis with ascites represents a condition in which kidney function critically depends on PGs. If a decline of renal function in cirrhotic patients is the result of the use of NSAIDs, withdrawal of treatment should usually be sufficient to improve renal function [26]. Animals with carbon tetrachloride-induced cirrhosis and ascites receiving NSAIDs or the selective COX-1 inhibitor SC-506, but not those receiving the selective COX-2 inhibitor celecoxib developed a severe impairment in renal function. These data indicate that COX-1- but not COX-2-derived PGs are involved in the homeostasis of kidney function in advanced cirrhosis [27,28,29,30]. In 28 nonazotemic patients with cirrhosis and ascites, short-term treatment with naproxen (500 mg every 12 hours for a total of five doses) but not administration of celecoxib (200 mg every 12 hours for a total of five doses) caused a significant reduction in GFR, renal plasma flow, urinary PGE_2_ excretion and suppression of the diuretic and natriuretic responses to furosemide. It was concluded that selective COX-2 inhibitors may be safer than nonselective NSAIDs in this patient population [31]. Some concerns were raised about the interpretation of the results. It was argued that the study was performed under tightly controlled conditions, as patients were hospitalized, diuretics were withheld, a salt-restricted diet was administered and the kidney function was probably influenced by the care provided during study hospitalization. In addition, reporting only average renal function before and after treatment may mask effects of drug toxicity in some patients [32]. These concerns are supported by a study of Guevara *et al.* [33] where mean GFR did not significantly change before and after treatment with celecoxib. However, four out of nine patients with cirrhosis and ascites showed a decrease greater than 20% in GFR after celecoxib. In contrast, no patient with cirrhosis and ascites in the study of Clària *et al.* [34] treated with celecoxib developed a significant (greater than 20%) decrease in GFR. The reasons for the different findings remain unclear. Previous studies have already shown that the administration of NSAIDs to patients with cirrhosis, ascites, and high plasma renin activity and norepinephrine is associated with a reduction in renal perfusion and GFR and ARF [35,36,37,38,39,40]. This effect, however, does not occur in patients with compensated cirrhosis or with ascites and normal plasma renin activity and norepinephrine indicating that increased renal synthesis of PGs in decompensated cirrhosis with ascites is a homeostatic response related to the activation of the endogenous vasoconstrictor system in order to maintain renal hemodynamics [35,36,37,38,39,40]. Data on the long-term safety of selective COX-2 inhibitors in cirrhosis are not available [31]. 

## 3. COX and the Renin-Angiotensin System

COX-2 activates the renin-angiotensin system, while an increased activity of the renin-angiotensin system inhibits COX-2. PGI_2_ and PGE_2_ increase potassium secretion primarily by stimulating the secretion of renin and activating the renin-angiotensin-aldosterone system [4]. Macula densa sensing of tubule NaCl concentration at the distal end of the loop of Henle serves as a primary regulatory step in renin secretion and tubuloglomerular feedback (TGF) [41,42]. Both TGF and renal renin production and release are modulated by PGs derived from the macula densa [43,44,45,46]. PG induced juxtaglomerular renin release is mediated via COX-2. In the other hand, COX-2 inhibitors inhibit renin production and secretion [46,47,48,49,50,51,52]. In addition, in mice with genetic deletion of COX-2, ACE inhibitors or low-salt diet failed to increase renal renin expression (in contrast to wild type mice), while renal renin expression was comparable between COX-1 null and wild type mice under these conditions [51,53,54]. Increased macula densa COX-2 expression in high-renin states, such as salt restriction, volume depletion, and renovascular hypertension [44,46,51] is mediated, at least in part, by nitric oxide [53].

Angiotensin-converting enzyme (ACE) inhibitors or angiotensin II receptor subtype I antagonists increase the expression of COX-2 in the kidney [55]. The feedback effects of angiotensin II on COX-2 are mediated via nitric oxide synthase-1 (neuronal nitric oxide synthase) [56,57]. In addition, mitogen-activated protein kinases (MAPKs) and, in particular, p38 are important for regulating COX-2 expression in the renal cortex. Low chloride concentrations significantly increase COX-2 and phosphorylated p38 expression [58].

## 4. COX-2 Inhibition and Sodium Retention

Expression by cortical COX-2 is increased by:

- sodium depletion
- renal artery stenosis
- aortic coarctation
- renal ablation
- loop diuretics
- Barter’s syndrome
- congestive heart failure [55].

In renal medullary interstitial cells both hypertonic and water-deprived conditions result in NF-κB driven COX-2 expression [59] suggesting that COX-2 selective inhibitors may render the medullary region of the kidney susceptible to cell death under these conditions [55].

Sodium retention is a well-described feature of all nonselective NSAIDs due to inhibition of COX-2 by these drugs. Therefore, it is predictable that COX-2 selective inhibitors may have similar effects [24,60,61]. In rats, rofecoxib, celecoxib, diclofenac and flurbiprofen but not meloxicam given orally once daily for 4 days caused a significant decrease in urinary sodium and potassium excretion as compared to placebo. NSAIDs administered orally to rats for four days had a transient and time dependent effect on the urinary excretion of electrolytes independent of COX-2-COX-1 selectivity [62]. In this animal study, meloxican did not affect sodium or potassium excretion rates, probably due to the low concentrations of meloxicam in the kidney [63]. However, these findings are limited by the fact that only one dose level for each NSAID was investigated [62]. In addition, clinical data are needed conforming the potential advantage of meloxicam in comparison to other COX-2 inhibitors. Interventional studies in elderly patients showed that selective COX-2 inhibitors have effects on both renal hemodynamics and sodium homeostasis that are quantitatively and qualitatively similar to those of nonselective NSAIDs [55]. Both coxibs and traditional NSAIDs can procedure impairment of kidney function, sodium retention with hypertension and peripheral edema, hyperkalemia and papillary necrosis [64]. In elderly subjects receiving a normal-salt diet, coxibs did not differ from naproxen in influencing sodium excretion, blood pressure, kidney function or weight changes [65]. No differences were found between indomethacin and coxibs with respect to proteinuria and kidney function in patients with amyloidosis secondary to rheumatic diseases [66]. Etoricoxib, a coxib of the second generation, also displayed dose-dependent renal adverse events similar to traditional NSAIDs [67]. COX-2 knockdown mice with profound and specific COX-2 inhibition displayed minimal signs of renal dysfunction but increased thrombotic activity [68], supporting the hypothesis that individuals taking coxibs could be predisposed to increased thrombotic tendency.

## 5. COX-2 and Renal Development

COX-2 is expressed constitutively not only in the adult but also in the fetal kidney [5,69,70,71]. COX-2 dependent PG formation is necessary for normal renal development. COX-2 deficient mice exhibit renal dysgenesis [72,73]. In contrast, gene knockout studies showed that COX-1 disruption does not interfere with normal renal development [74]. Administration of a COX-2 selective inhibitor during pregnancy significantly impaired development of the renal cortex and reduced glomerular diameter in both mice and rats, identical to transgenic COX-2^−/−^ mice, while administration of a COX-1 selective inhibitor did not affect renal development. Prostanoids or other products resulting from COX-2 activity in the macula densa may act in a paracrine manner to influence glomerular development [75].

## 6. COX and Glomerular Diseases

Chemokines such as monocyte chemo-attractant protein-1 (MCP-1) are expressed in glomeruli of animals and humans with glomerulonephritis. MCP-1 is involved in the monocyte/macrophage infiltration into glomeruli and the renal interstitium [76,77,78,79]. Mesangial cell production and release of MCP-1 is stimulated by cytokines and growth factors [80,81,82], while dexamethasone [83] or PGE [84] reduces the glomerular MCP-1 expression, suggesting that endogenously formed PGs can modulate the formation of MCP-1 and influence the clinical outcome of experimental glomerulonephritis. Schneider *et al.* [85] examined the renal effects of COX-2 selective inhibitors *versus* indomethacin in two different models of glomerulonephritis: anti-thymocyte serum induced mesangioproliferative glomerulonephritis and anti-glomerular basement antibody induced glomerulonephritis. All NSAIDs augmented the glomerular production of MCP-1 and RANTES suggesting that endogenous PGs normally suppress renal chemokines formation. Increased monocyte/macrophage infiltration was observed only in those animals treated with indomethacin suggesting also a role for COX-1 products in suppressing renal inflammation [85].

Pro-inflammatory agents such as interleukin-1ß [86] and lipopolysaccharide (LPS) [87] induce PGE_2_ by COX-2 indicating that COX-2 generated PGE_2_ plays an important role in inflammatory processes, such as glomerulonephritis [88,89,90]. In experimentally induced immune-mediated glomerulonephritis, PGE decreases damage of the kidney through reduction of glomerular immune complex formation, through reduction of inflammatory cell infiltration and through reduction of deposition of extracellular matrix products [91,92,93]. Prostaglandin EP2 and EP4 receptors modulate the expression of MCP-1 in response to LPS-induced renal glomerular inflammation: Overexpression of EP2 and EP4 decreases MCP-1 expression, while the down-regulation of EP2 and EP4 receptors results in an imbalance in the inflammatory state of mesangial cells. It was concluded that COX products may participate in the monocyte/macrophage clearing and in the healing process in glomerulonephritis [94].

Certain NSAIDs, such as sulindac, ibuprofen, and flurbiprofen may exert anti-inflammatory effects independently of COX activity and prostaglandin synthesis. Those anti-inflammatory effects are mediated by inhibition of certain transcription factors such as activator protein 1 and nuclear factor-κB and/or by alterations in the activity of IκB kinase, mitogen-activated protein kinase, and cyclin-dependent kinase [95]. NSAIDs, especially indomethacin, have the potential to attenuate proteinuria in different types of glomerulonephritis [96,97,98] and nephrotic syndrome [99,100]. Podocytes play an important role in the maintenance of the integrity of the split diaphragm that regulates passage of macromolecules from plasma to the urinary space. In murine podocytes, expression of MCP-1 in response to tumor necrosis factor-α (TNF-α) was suppressed by indomethacin but not by ibuprofen. Indomethacin induced in podocytes the 78-kDa glucose-regulated protein (GRP78), a marker of unfolded protein response (UPR), the expression of CHOP, another endogenous indicator of UPR and the repression of endoplasmic reticulum stress-responsive alkaline phosphatase, an exogenous indicator of UPR. However, neither ibuprofen nor aspirin or sulindac induced UPR, indicating that indomethacin inhibits inflammatory responses of cells not only by COX inhibition but also by inhibition of TNF-α-triggered activation of nuclear factor-KappaB (NKF-κB) via induction of UPR [101].

In very rare cases, NSAIDs may induce glomerular disease, such as membranous nephropathy which is clinically complicated by nephrotic syndrome. Not only renal transplant patients but also patients with different forms of glomerulonephritis (e.g., membranous nephropathy, focal segmental glomerulosclerosis, steroid-resistant minimal change nephropathy) may be treated with a calcineurin inhibitor. The kidney is vulnerable toward adverse effects of the calcineurin inhibitors cyclosporine A and tacrolimus, including decrease of GFR, tubular dysfunction, glomerulosclerosis, and renal interstitial fibrosis. In Wistar-Kyoto (WKY) rats treated with cyclosporine A (15 mg/kg per day) or tacrolimus (5 mg/kg per day) for seven days each, both drugs markedly lowered renal COX-2 expression while COX-1 expression remained unaltered. Cyclosporine A blunted the increase of renocortical COX-2 expression in response to low salt intake or the combination of low salt-intake with an ACE inhibitor, while renin secretion and renin gene expression were enhanced. These data indicate that calcineurin inhibitors selectively suppress renal COX-2 expression without attenuating the regulation of the renin system [102]. Cyclosporine A may aggravate renal adverse events associated with the use of NSAIDs [103]. Both cyclosporine A and tacrolimus therapy causes afferent renovasoconstriction which is aggravated by NSAIDs resulting in further decline in renal blood flow and GFR. Therefore, in renal transplant recipients on immunosuppression with cyclosporine A or tacrolimus suffering from chronic pain, NSAIDs should be replaced by metamizole, acetaminophen, tramadol and/or steroids.

## 7. COX and Diabetic Nephropathy

Diabetic nephropathy is a leading cause of ESRD. Renal hyperfiltration is a risk factor for progression of diabetic nephropathy. COX-2 is an important determinant of renal hemodynamic function in subjects with type 1 diabetes. Experimental models of diabetes revealed that COX-2 expression is increased in the macula densa in this condition and is associated with enhanced production of vasodilatory PGs, renin-angiotensin system activation, and renal hyperfiltration. In diabetic rats, hyperglycemia-associated PG production and hyperfiltration were blunted using COX-2 inhibition [104]. In normotensive, normoalbuminuric adolescents and young adults with type 1 diabetes, COX-2 inhibition resulted in a significant decline in GFR in the hyperfiltration group but increased GFR in the normofiltration group [105], indicating that the renal hemodynamic response to COX-2 inhibition is dependent on GFR. Thus, not only the renin-angiotensin system but also COX-2 contributes to the hyperfiltration state in diabetes. COX-2 inhibition decreases proteinuria and retards progressive renal injury in rats [106]. In patients with type 1 diabetes, short-term indomethacin therapy reduced urinary albumin excretion without altering GFR or blood pressure [107]. In patients with diabetic nephropathy, a single oral dose of ibuprofen reduced GFR and renal blood flow after two hours but did not influence blood pressure or fractional excretion of sodium [108].

There are gender differences in the renal and peripheral hemodynamic response to COX-2 inhibition and intrarenal interaction between COX-2 and the renin-angiotensin system. In young women with uncomplicated type 1 diabetes mellitus, COX-2 inhibition (200 mg celecoxib daily for 14 days) was associated with significant increases in filtration fraction (an indirect measure for the intraglomerular pressure) and vascular resistance, and also associated with a significant decline in renal blood flow as compared to men. The decline in GFR in response to a graded angiotensin II infusion was abolished by COX-2 inhibition in diabetic women but not in diabetic men, indicating augmented female prostanoid dependence of renal hemodynamic in type 1 diabetes [109].

Flow-mediated dilation (FMD) in the brachial artery is significantly higher in normofiltering *versus* hyperfiltering subjects with type 1 diabetes. In response to COX-2 inhibition during clamped euglycemia, FMD declined significantly in normofiltering but not in hyperfiltering subjects. This effect was abolished by hyperglycemia. It was concluded that systemic hemodynamic function, including the response to COX-2 inhibition, is related to renal filtration status in patients with type 1 diabetes, probably as a results of general endothelial dysfunction [110].

In a non-obese insulin-dependent mouse model of spontaneous type 1 diabetes, low dose ibuprofen (1 mg/kg/day in the drinking water) or COX-2 specific inhibitor NS-398 was administered from 20 weeks of age and continued until 36 weeks. Ibuprofen reduced GFR and albuminuria, while blood pressure was not affected. Both the cortical cyclin-dependent kinase inhibitor p27 (an important regulator of renal and glomerular hypertrophy) and renal fibronectin were increased in the diabetic animals but attenuated by ibuprofen. Thus, chronic low-dose ibuprofen therapy may be beneficial at the onset of diabetic nephropathy [111]. Cheng *et al.* [112] examined a model of diabetes and hypertension, in which streptozotocin diabetes was combined with deoxycorticosterone (DOCA)-salt treatment. In this model, glomerular injury progressed at a faster rate than in animals with diabetes or DOCA-salt alone. The renal expression of COX-2 was increased, along with that of fibronectin, transforming growth factor-beta and plasminogen-activator inhibitor. The administration of the selective COX-2 inhibitor SC58236 to these rats reduced the expression of these mediators and the development of glomerulosclerosis [112].

Angiotensin II processes two main receptors, the angiotensin II type 1 receptor (AT1R) involved in the vasoconstrictor and growth properties of angiotensin II and the angiotensin II type 2 receptor (AT2R) involved in vasorelaxation. Impaired vasodilation on Zucker diabetic fatty rats returned to control after superoxide reduction, COX-2 inhibition or TXA_2_ synthesis inhibition, indicating that in type 2 diabetic animals, reactive oxygen species and COX-2-derived TXA_2_ reduce AT2R-induced vasorelaxation [113]. By far more clinical studies are needed to define benefits and risks of COX-2 inhibitors in type 1 and type 2 diabetics.

## 8. COX in Ureteral Obstruction and Lithium Nephropathy

COX activity contributes to renal function changes immediately after onset of ureteral obstruction [114,115,116]. Expression of COX-2, but not COX-1, is markedly increased in the inner medulla in response to unilateral and bilateral ureteral obstruction [114,115,117]. Administration of a selective COX-2 inhibitor prevents downregulation of aquaporin-2 (AQP2) in inner medullary collecting ducts, abolishes the increase in urinary excretion of the PGE_2_ and PGI_2_ metabolite 6-keto-PGF_1α_, and attenuates the polyuria observed typically during the first day after the release of bilateral ureteral obstruction induced for 24 hours [115,116]. Bilateral ureteral obstruction caused also an increase of PGE_2_, PGF_2α_, 6-keto-PGF_1α_ and TXB_2_ in inner medulla tissue which was prevented by the administration of a COX-2 inhibitor [118]. Water loading causes a decrease in AQP2 expression, while dehydration increases the AQP2 levels [119]. Changes in AQP2 expression also occur in a variety of pathological conditions associated with either excess water loss or water retention. Dehydration-induced increase in AQP2 abundance is blocked by NSAIDs [120]. This may be of particular significance in subjects, who are prone to dehydration, such as older and critically ill patients. Ureteral obstruction activates the intrarenal renin-angiotensin-system and increases intrarenal angiotensin II generation [121,122,123]. Angiotensin II receptor type 1A (AT1R) blockade significantly reduces COX-2 abundance in the postobstructed kidney and also attenuates the angiotensin II-mediated downregulation of aquaporin water channels and key renal sodium transporters in response to urinary tract obstruction [124]. However, AT1R-mediated AQP2 regulation in the postobstructed kidney collecting duct is independent of COX-2 induction [125]. 

The antidiuretic action of vasopressin depends on the exocytic insertion of AQP2 water channels from a store in intracellular vesicles to the apical plasma membrane of collecting duct principal cells, the so-called “shuttle” mechanism. Indomethacin markedly reduces the expression of AQP2 water channels in the collecting duct but enhances the shuttling of AQP2. The increased shuttling of APQ2 results in diminished urine volume. The altered urinary concentration ability and body water balance associated with the use of NSAIDs may in part be causally related with the alteration of AQP2 [126]. Lithium treatment is one of the major causes of the acquired form of nephrogenic diabetes insipidus (NDI), a clinical syndrome in which the kidney is unable to concentrate urine despite normal or elevated concentrations of the antidiuretic hormone arginine vasopressin. In lithium-induced NDI rat models, downregulation of AQP2 has been demonstrated. For the treatment of NDI, NSAIDs or coxibs have been useful [127,128,129]. The upregulation of AQP2 and the Na-K-2Cl inhibition underlies the therapeutic mechanisms by which COX-2 inhibitors enhance antidiuresis in patients with NDI [130].

## 9. NSAIDs and Blood Pressure

PGs contributed to blood pressure homeostasis via their effects on vascular tone and on renal fluid and electrolyte transport. NSAIDs cause little or no increase in blood pressure in normotensive individuals. However, NSAIDs and COX-2 inhibitors may increase systemic blood pressure in hypertensive persons and/or undermine blood pressure control with antihypertensive drugs [131,132]. In rodents, COX-1 deletion causes natriuresis and enhances sensitivity to ACE inhibitors. Deficiency of COX-1 reduces blood pressure despite activation of the renin-angiotensin system [54]. Both pharmacological inhibition and genetic deletion of COX-1 abolish the hypertensive response to angiotensin II [133,134]. In contrast, deletion or inhibition of COX-2 reduces renal medullary blood flow and sodium excretion, increases the vasoconstrictive response to angiotensin II [134] and elevates blood pressure [135]. COX-2 deficient mice on a normal diet exhibited systolic hypertension, whereas blood pressure was unaltered in COX-1 > COX-2 mice *versus* wild-type littermates. However, in response to a high salt diet, also COX-1 > COX-2 mice developed hypertension. These data indicate that COX-1 can rescue COX-2 under physiological conditions but is unable to compensate for the absence of COX-2 in regulating blood pressure homeostasis in the face of a high salt challenge. COX-2-dependent capacity of the renal medulla to generate vasodilatory PGs such as PGE_2_ and PGI_2_ was also assessed in these animals. While high salt diet augmented the capacity to generate renal medullary PGs in wild type mice, this capacity was attenuated in the case of PGI_2_ in both mutant strains. Thus, COX-2 cannot compensate for the failure to augment PGI_2_ formation in response to a high salt diet [136].

COX-2 selective inhibitors have effects on blood pressure that are similar to those of nonselective NSAIDs [137,138,139,140,141,142,143]. In contrast, the cardiorenal safety database from the Celecoxib Long-term Arthritis Safety Study (CLASS) indicates that a supratherapeutic dose (400 mg b.i.d.) of celecoxib was associated with an improved cardiorenal safety profile compared with standard doses of either ibuprofen or diclofenac. Celecoxib was associated with a lower incidence of hypertension or edema than ibuprofen. The celecoxib group had significantly fewer initiations of antihypertensives than patients taking ibuprofen. Systolic blood pressure increase >20 mmHg and above 140 mmHg occurred significantly less often with celecoxib as compared to ibuprofen or diclofenac [144]. In a subgroup of patients with prerenal azotemia, significantly fewer patients taking celecoxib exhibited clinically important reductions in renal function (3.7%) as compared to diclofenac (7.3%) or ibuprofen (7.3%). It was concluded that celecoxib may frequently be a more suitable treatment of chronic pain and inflammation than nonselective NSAIDs in patients with compromised renal function [144]. In the study by Sowers *et al.* (145), patients with hypertension, osteoarthritis, and type 2 diabetes mellitus were randomly assigned to treatment with 200 mg of celecoxib daily (n = 136), 25 mg of rofecoxib once daily (n = 138), or 500 mg of naproxen twice daily (n = 130) for 12 weeks. The blood pressure difference between rofecoxib and celecoxib was 3.78 mmHg (p = 0.005), and between rofecoxib and naproxen 3.85 mmHg (p = 0.005). The proportion of patients with controlled hypertension at baseline who developed ambulatory hypertension by week 6 was significantly greater with rofecoxib (30%) than with celecoxib (16%) but not greater than with naproxen (19%). 

Risk for cardiovascular death is high among patients with rheumatoid arthritis [146]. Because of the increased risk of thrombotic events, the manufacturers of rofecoxib (September 2004) and valdecoxib (April 2005) withdrew their products. However, no selective COX-2 inhibitor is risk free. The Adenoma Prevention with Celecoxib (APC) trial using 400 to 800 mg daily doses of celecoxib had been prematurely terminated owing to a significant excess of cardiovascular death, myocardial infarction, and stroke [147]. Celecoxib in typical 100 to 200 mg daily doses has a lower risk of cardiovascular toxic effects as compared to rofecoxib or valdecoxib. NSAIDs were suggested to have a cardioprotective effect by inhibition of platelet aggregation through inhibition of COX-1. However, inhibition of vascular COX-2 in the presence of inadequate inhibition of platelet COX-1 results in enhanced risk of adverse cardiovascular events including myocardial infarction [148]. In addition, inhibition PG synthesis may cause hypertension, but COX-2 selectivity alone does not define the cardiovascular risk associated with NSAIDs [149]. Celecoxib but not other coxibs or diclofenac inhibits calcium responses in vascular smooth muscle cells by enhancing voltage-gated KCNQ5 K^+^- and suppressing Ca^+^- channels, which ultimately reduces vascular tone, independent of its COX-2 inhibitory actions. These COX-2-independent actions of celecoxib may offset what would otherwise be a detrimental increases in vasoconstriction mediated by COX-2 inhibition and explain the differential risk of cardiovascular events in patients taking different drugs of this class [150,151]. Dilation of blood vessels and reduction in systemic blood pressure by celecoxib suggest that the reduced work load on the heart may counteract any other deleterious effects of this class of drugs [152]. 

The CLASS and VIGOR (Vioxx Gastrointestinal Outcomes Research) studies provided evidence for increased blood pressure in a minority of subjects less than or equal to (celecoxib) or greater than (rofecoxib) the NSAID comparators [137,138]. The Successive Celecoxib Efficacy and Safety Studies (SUCCESS) VI and VII compared the renal safety in older hypertensive patients with osteoarthritis and found that at week 6, rofecoxib was more likely to increase the systolic blood pressure than celecoxib [22,23]. Celecoxib (200 mg once a day) caused less development of peripheral edema (4.9% *versus* 9.5%; P = 0.014) and less loss of blood pressure control (11% *versus* 17%; P = 0.032) than rofecoxib [134], but invalidity of dose comparison and the imbalance in the number of patients who received ACE inhibitors between both groups of this study have been criticized [55]. In a meta-analysis of 114 clinical trials involving 116,094 patients, rofecoxib treatment was associated with peripheral edema (RR 1.43, 95% CI:1.23–1.66), hypertension (RR 1.55, 95% CI:1.29–1.85), and renal dysfunction (RR 2.31, 95% CI:1.05–5.07), while patients on celecoxib treatment did not differ from controls. The RRs for renal dysfunction and peripheral edema were 0.61 (95% CI:0.40–0.94) and 1.09 (95% CI:091–1.31) in celecoxib users, respectively [153]. The findings suggest that there does not appear to be a class effect in terms of renal adverse events with selective COX-2 inhibitors [154]. In young and elderly normotensive subjects on celecoxib (200mg b.i.d. for 2 weeks), no significant effects on parameters of the renin-angiotensin-aldosterone system, kidney function and blood pressure have been observed [15], while in healthy volunteers with mild volume depletion, COX-2 inhibition caused a 65% decrease in plasma renin activity (p = 0.008), which was antagonized by the combined intake of celecoxib and irbesartan. Neither GFR nor renal sodium and potassium excretion was influenced by a single dose of 400 mg celecoxib intake alone or combined with 150mg irbesartan [155]. Therefore, PGs that increase renin production in response to ACE inhibition are not derived from COX-1 [156].

The incidence rate of renal side effects associated with the use of selective and nonselective COX-2 inhibitors is low in otherwise healthy subjects but can get as high as 20% in high risk patients [157]. In addition, patients who take NSAID, are often afflicted with other disease, need other medications, and may have various risk factors purported to influence the side effects of NSAIDs [158]. In patients with hypertension, increased activation of the renin-angiotensin and sympathetic nervous system may cause subsequent release of vasodilator prostaglandins from the kidney, which act locally to lessen the degree of renal hypoperfusion [159]. In case that this compensatory mechanism is inhibited by NSAIDs, the increase in renal and systemic vascular resistance can cause an elevation of blood pressure [160]. Nonselective NSAIDs may antagonize the blood pressure-lowering effect of antihypertensive medications, including diuretics, ACE inhibitors, and β-blockers [139,161,162]. In a controlled clinical trial in patients with mild to moderate hypertension receiving β-blockers plus diuretics, three weeks treatment with ibuprofen (1,200 mg/day) caused an increase in supine blood pressure by 5.3 mmHg and in sitting mean arterial pressure of 5.8 mmHg as compared to placebo [163]. An increase in blood pressure was also noted in a placebo-controlled clinical trial in patients receiving hydrochlorothazide and 1,800 mg/day ibuprofen [164]. Combinations of ACE inhibitors/angiotensin receptor antagonists, diuretics and NSAIDs may impair renal function, particularly in the elderly [165]. Seelig *et al.* [166] performed a search of the records of 2278 patients with NSAIDs, 328 with ACE inhibitors, and 162 with both. No nephrotoxicity was found in conjunction with monotherapy but three cases of reversible ARF were observed in conjunction with the combination of NSAID and ACE inhibitor. Of 27 patients with ARF due an ACE inhibitor, six patients were also on a NSAID [167]. A patient aged 85 years developed life-threatening hyperkalemia during treatment with the combination of an ACE inhibitor and a NSAID [168]. Two of 12 elderly subjects treated with the co-prescription of ACE inhibitors, diuretics and NSAIDs developed ARF, and four patients showed deterioration in renal function, which returned to normal after stopping the NSAID in three and the ACE inhibitor in one [169]. Therefore, care is necessary to balance the demonstrated advantages of these medications against the risk of inducing kidney failure [170]. 

Decrease in diuretic response was reported when furosemide was simultaneously administered with indomethacin in humans [171]. Indomethacin inhibited the saluretic and diuretic response to furosemide both in adult and newborn rats. Inhibitory interaction between indomethacin and furosemide was achieved at approximately 10-fold lower concentrations in the newborne than in the adult rats, suggesting that the neonate kidney is more sensitive to the action of these drugs than the adult kidney [172]. Indomethacin or meclofenamate blunted the response to furosemide on sodium and chloride transport [173], suggesting that the drugs interact at the Na-K-2Cl cotransporter. Since COX-2-derived PGE_2_ is found primarily on the thick ascending limp of the loop of Henle, NSAIDs can lessen response to loop-acting diuretics by as much as 20% (or even more in patients likely to retain sodium, such as in those with congestive heart failure or cirrhosis) [24]. COX inhibition by diclofenac or rofecoxib reduces significantly the hydrochlorothiazide-induced urinary sodium excretion, while urinary potassium excretion is not affected [174]. The COX-2 inhibitor rofecoxib blunts the diuretic-induced increase in renal excretion of prostanoid, indicating an effective blockade of COX-2. Rofecoxib dose-dependently attenuates diuresis and saluresis, and also the stimulation of the renin system induced by furosemide. It completely reverses diuresis and saluresis, and prevented the increase of plasma renin activity induced by hydrochlorothiazide [175]. Cyclosporin attenuates furosemide-induced natriuresis, likely by inhibition of COX-2-mediated natriuresis. A combination of cyclosporine with rofecoxib has no additive effects on PGE_2_ formation, natriuresis and diuresis [176]. NSAIDs contribute to resistant hypertension [177]. Interestingly, the increase in blood pressure by selective COX-2 inhibitors can be reduced or even prevented by salt deprivation [178,179]. Rofecoxib dose dependently increased systolic blood pressure and decreased 6-keto-PGF_1α_ in both normotensive WKY rats and in spontaneously hypertensive rats (SHR) fed normal (0.6% NaCl, wt wt^−1^) or high salt (8% NaCl, wt wt^−1^) but not in both rat strains on low salt (0.02% NaCl, wt wt^−1^) intake suggesting that chronic inhibition of COX-2 causes an increase of blood pressure that depends on prostacyclin synthesis. This increase is independent on genetic predisposition and can be prevented by salt deprivation [178]. However, COX-1-derived, but not COX-2-derived, prostanoids are of relevance for the regulation of the renin system by salt intake [180]. 

COX-2-inhibition enhances the pressure effect of angiotensin II [181]. In patients with essential hypertension, even high doses of celecoxib (400 mg/day) did not cause any alteration of the antihypertensive effect of lisinopril [145,182]. ACE inhibitors and angiotensin II receptor blockers are efferent renovasodilators and may cause functional, but reversible, renal insufficiency, which may worsen with NSAIDs by inducing afferent renovasoconstriction. Therefore, lowering of the dose of the NSAIDs as much as possible, lowering of salt intake, retitration of the antihypertensive and calcium channel blockers have been recommended when treating hypertension in a patient taking an NSAID. Another strategy is to use a non-NSAID, such as tramadol or aspirin [6]. Interestingly, patients with rheumatoid arthritis or osteoarthritis and cardiorenal risk factors such as hypertension, congestive heart failure, edema, renal impairment, and advanced age were more likely to receive a coxib than other NSAIDs [183]. 

Clinical studies are needed to confirm the above mentioned favourable *in vitro* effects of celecoxib [150,151] on the reduction of vascular tone in hypertensive patients. The increase of blood pressure observed in subjects on NSAIDs depends of the age of the patients, the NSAIDs dose used and the co-medication prescribed to control elevated blood pressure. For example, indomethacin raises blood pressure in elderly patients whose blood pressure had been controlled on an ACE inhibitor but has little or no effect on blood pressure in patients controlled on amlodipine or felodipine [184]. In people controlled on verapamil there was no significant rise in blood pressure by ibuprofen or naproxen [185]. In a meta-analysis undertaken by Johnson *et al.* [186], it appeared that the increase of blood pressure by NSAIDs was greater in people on β-blocking drugs than in those on diuretics or vasodilators. In a group of elderly, normotensive people, ibuprofen elevated blood pressure, while it had no effect in a young group [187]. As discussed before calcium channel blocking drugs are recommended and/or diuretics, if NSAIDs primarily cause a rise in blood pressure due to sodium retention [184].

Acute renal artery stenosis induced by an unilateral renal artery clip causes ipsilaterally an acute upregulation and contralaterally a downregulation of juxtaglomerular COX-2 expression. Plasma renin activity and renocortical renin mRNA in the clipped kidney increased markedly, while in the contralateral kidney renin mRNA decreased to 50% of normal values. Treatment of the animals with the COX-2 blocker celecoxib (40 mg/kg/day) did not chage plasma renin activity nor renin mRNA either in the clipped or in the contralateral intact kidney. It was concluded that COX-2-derived prostaglandins were not involved in the control of renin expression during renal hypoperfusion [188]. However, in hypoperfused kidneys induced by aortic coarction, the COX-2 inhibitor SC-58236 almost blunted the rises of plasma renin activity and of renin mRNA in the kidneys [189]. Isoproterenol or unilateral renal artery clipping for two days increases plasma renin activity and renin mRNA in the kidneys to similar levels in rats treated with both the vehicle or the COX-2 inhibitor SC-58236 after two days, while pretreatment with SC-58236 for five days reduced the absolute increase in plasma renin activity and renin mRNA. It was concluded that COX-2 activity determines the set point for the activity of the renin system in rat kidneys [190].

## 10. NSAIDs and Acute Renal Failure

When possible, selective and nonselective NSAIDs should be avoided in patients with CKD, congestive heart failure, or liver cirrhosis to prevent ARF [191]. There is some evidence to support increased incidence of adverse effects with increased dosing of selective and nonselective NSAIDs [192]. Some medications, such as ACE inhibitors, angiotensin II-receptor blockers and β-blockers, may increase NSAID-related renal complications.

High acute dose of NSAIDs, have been implicated as causes of ARF, particularly in the elderly [193,194]. Some reported cases of ARF after initiation of NSAID therapy include apparently healthy subjects [3,4]. Case reports have documented ARF in association with both celecoxib and rofecoxib [142,195]. Of 1799 frail elderly patients hospitalized with community-acquired ARF 18.1% were current users of prescribed NSAIDs [193]. In this study, a strong dose-dependent increase of risk for ARF was observed in those 35% subjects taking ibuprofen as NSAIDs: Odds ratios associated with dosages of ≤1,200 mg/day, >1,200- <2,400 mg/day, and ≥2,400 mg/day were 0.94, 1.89, and 2.32, respectively. An explanation for this dose-dependent finding was not provided. Patients submitted with ARF had a greater comorbidity, and also a greater use of diuretics and ACE inhibitors than the respective controls [193]. Endogenous angiotensin II is an inhibitor of COX-2 expression in the macula densa. Conversely, ACE inhibition and angiotensin II type 1 receptor blockade potently upregulates COX-2 [196] and thus may exacerbate NSAID related renal functions [197].

NSAID treatment is a risk factor for contrast media induced nephropathy (CIN), mostly defined as a relative increase of serum creatinine by ≥25% or a decrease of GFR by ≥25% within 24–72 hours after contrast media exposure. CIN is a common complication in high risk patients such as those with CKD and diabetes mellitus. Radiocontrast agents cause vasoconstriction of the vas afferens and may aggravate NSAID induced decrease in renal blood flow, GFR and intraglomerular pressure, particularly in risk patients treated with an ACE inhibitor or angiotensin II blocker. It is, therefore, recommended to discontinue selective or nonselective NSAID therapy 48 hours before administration of radiocontrast agents in those patients. Weisbord *et al.* [198], however, reported that 67 of 660 (10.2%) patients with GFR less than 60 mL/min/1.73 m² undergoing procedures with intravenous radiocontrast were prescribed NSAIDs but only three patients were instructed to discontinue the medication.

Various biochemical abnormalities produced in the kidney in response to the administration of indomethacin include oxidative damage and impairment of structure and function of mitochondria mediated through the production of free radicals [199]. Indomethacin induces also impairment in structure and function of brush border membranes in the kidney mediated by free radicals and the activation of phospholipases [200]. Sepsis and septic shock are important risk factors for acute renal failure due to alterations in glomerular hemodynamics. Endotoxemia causes also a time- and dose-dependent decrease of the renocortical expression of the organic anion transporters OAT1 and OAT3 that paralleled the increased renocortical COX-2 expression and PGE_2_ formation. OATs are also downregulated during ischemia/reperfusion-induced ARF and ureteral obstruction, conditions under which renal COX-2 expression is increased. Pretreatment with the COX-2 inhibitor parecoxib attenuates not only OAT1 and OAT3 gene repression in the rat kidney following endotoxin treatment but also the fall in creatinine clearance and para-aminohippurate clearance [201]. 

As any drug, NSAIDs may cause ARF due to acute interstitial nephritis as a result of allergic hypersensitivity reaction few days after initiation of NSAID therapy. In this case, kidney function usually recover when traditional NSAIDs or coxibs are discontinued. If not, prednisone therapy (1 mg/kg per day) should be considered. Long-term NSAID use may result in chronic interstitial nephritis with interstitial fibrosis and chronic renal dysfunction.

## 11. NSAIDs and Risk for Chronic Kidney Disease

Analgesic nephropathy is a slowly progressive chronic kidney disease resulting from daily use for many years of preparations containing at least two analgesics (e.g., aspirin, acetaminophen, phenacetin or pyrazolones) in combination with central-acting dependence-inducing substances, such as caffeine, codein, and/or barbiturates. Analgesic nephropathy is characterized by capillary sclerosis, renal cortical atrophy, chronic interstitial nephritis and/or papillary sclerosis/necrosis/calcifications. In a number of patients with analgesic nephropathy, the uroepithelia can develop transitional cell carcinoma. Analgesic nephropathy can be accurately diagnosed or excluded by computed tomography scanning without contrast media [202]. Even if renal papillary necrosis occurs in patients with analgetic nephropathy, traditional NSAIDs including ibuprofen [203], tolmetin [204], indomethacin [205], benoxaprofen [206], and naproxen [207,208], have been also reported to cause renal papillary necrosis.

No association between regular use of analgesics such as acetaminophen, aspirin, or NSAIDs and chronic renal dysfunction has been observed [209,210], while other studies showed increased risk [211,212,213,214,215]. A case-control study reported a 2-fold increased risk of end-stage renal disease among individuals with lifetime use of more than 1,000 acetaminophen pills and an 8-fold increased risk among those with a lifetime cumulative dose of more than 5,000 NSAID pills [216]. In contrast, multivariable analyses performed in a total of 11 032 initially healthy men demonstrated that the relative risks of elevated creatinine level associated with intake of 2,500 or more analgesics pills were 0.83 for acetaminophen, 0.98 for aspirin, and 1.07 for other NSAIDs. No association was observed between analgesic use and reduced creatinine clearance. It was concluded that a moderate analgesic use in this cohort study of initially healthy men was not associated with increased risk of renal dysfunction [216]. A large case-control study found a greater than 2-fold increased risk of newly diagnosed chronic renal insufficiency for regular users of acetaminophen or aspirin but not for those using regularly NSAIDs [217]. In the Nurse’s Health Study, acetaminophen use was associated with an increased risk of GFR decline in 11 years, but aspirin and NSAID use not [218]. In contrast, some case-control studies found an association between NSAIDs and the risk of chronic renal dysfunction [215,219].

Lafrance and Miller [211] conducted a retrospective nested case-control study based on data from a cohort of 1.459.271 new NSAID user and identified 22.824 cases of acute kidney injury (AKI), defined as a creatinine increase of greater than 50%. The risk of AKI was found to be lower with more selective agents than with naproxen or other non-selective NSAIDs. For example, adjusted odds rations (ORs) were 0.96 (95% CI:0.63–1.47) for celecoxib, 1.13 (95% CI:0.63–2.05) for meloxicam, 1.11 (95% CI:0.84–1.48) for diclofenac, 1.53 (95% CI:1.05–2.23) for piroxicam, 1.61 (95% CI:1.12–2.30) for sulindac, 2.25 (95% CI:2.04–2.49) for ibuprofen, 1.72 (95% CI:1.52–1.95) for naproxen and 1.94 (95% CI:1.56–2.42) for indomethacin, respectively. The highest risk (OR = 2.90; 95% CI:2.62–3.22) for AKI was fond in patients using multiple NSAIDs. It was concluded that the risk of AKI is not homogenous among different NSAIDs and that more selective NSAIDs may present a better safety profile for AKI [220]. Only 2% will stop taking NSAIDs after developing renal complications [191].

Gooch *et al.* [221] determined the association between NSAID use and the progression of CKD in an elderly community-based cohort. A total of 10,184 subjects (mean age 76 years) were followed for a median of 2.75 years. High-dose NSAID users (upper decile of cumulative NSAID exposure) was associated with an increased risk for rapid CKD progression among subjects with a baseline mean GFR between 60 and 89 mL/min/1.73 m² without risk differential between selective and nonselective NSAID users [221]. Taken together, physicians should always prescribe the lowest effective dose of NSAIDs for the shortest possible time [222].

## 12. Future Developments

Adverse cardiovascular events associated with selective COX-2 inhibitor therapy has provided a strong stimulus for the development of NO-NSAIDs. NO (nitric oxide) exhibits beneficial cardiovascular effects such as vasodilation and inhibition of platelet aggregation [223]. NO-NSAIDs containing novel diazonium-diolate groups have the potential to theoretically release two molecules of NO with half-lives that correlate well with their pharmacological durations of actions avoiding nitrate tolerance [224]. In addition to NO-NSAIDs, dual COX/lipoxygenase (LOX) inhibitors and anti-TNF therapy represent novel approaches directed toward the development of effective anti-inflammatory therapy [223]. Naproxcinod is the first representative of the Cyclooxygenase Inhibiting Nitric Oxide Donator (CINOD) class of NSAIDs [225]. Clinical trial with this compound are ongoing in patients with osteoarthritis.

Among the three different isoforms of PGE synthase, m(membrane)PGES-1 is a promising novel therapeutic target [226]. MF63 [2-(6-cholor-1H-phenanthro[9,10-d]imidazol-2-yl]isophthalonitrile] is a potent and selective mPGES-1 inhibitor with anti-pyretic and anti-inflammatory properties comparable to selective or nonselective COX-2 inhibitors [227]. Renal safety, however, is uncertain [148]. A series of orally active EP4 antagonists are currently under investigation and display COX-2 inhibitor-like analgesic, anti-inflammatory and renal effects [228], directly blocking the receptor from functioning [222].

## 13. Conclusions

NSAIDs inhibit both COX-1 and COX-2, the rate limiting enzymes for the production of PGs and TX. Both isoenzymes are located within the kidney. Blockade of either or both of these enzymes may affect different renal functions. COX-2 derived PGs have profound effects on renal homeostasis suggesting that selective COX-2 inhibitors such as celecoxib may have the same potential for adverse renal effects as traditional NSAIDs, particularly in clinical situations associated with impairment of kidney function such as salt depletion, hypovolemia, liver cirrhosis, congestive heart failure, nephrotic syndrome and CKD. NSAIDs may induce sodium and fluid retention (particularly in the elderly) and increase blood pressure or aggravate an already existing hypertension. Dietary salt restriction, reduction in the NSAID dose, use of non-NSAID analgesics, treatment with calcium channel blocker (in order to reduce renal vasoconstriction) and/or diuretics (even if less effective in the presence of NSAIDs) are possible options for the patients who developed hypertension. Selective COX-2-inhibitor such as celecoxib may affect blood pressure less than traditional NSAIDs. These compounds may dose-dependently increase the risk for ARF, particularly in the elderly with high co-morbidity, and the use of the combination of ACE inhibitors/angiotensin II blockers, diuretics and NSAIDs. Whether regular intake of NSAIDs is a risk factor for end-stage renal disease is controversly discussed in the literature.
